# Compassion fatigue in healthcare providers: a scoping review

**DOI:** 10.1186/s12913-023-10356-3

**Published:** 2023-12-01

**Authors:** Anna Garnett, Lucy Hui, Christina Oleynikov, Sheila Boamah

**Affiliations:** 1 Arthur Labatt Family School of Nursing, Western University, London, ON Canada; 2 Medical Sciences, Western University, London, ON Canada; 3 School of Nursing, McMaster University, Hamilton, ON Canada

**Keywords:** Compassion fatigue, Healthcare provider, COVID-19, Psychological health, Well-being, Patient care

## Abstract

The detrimental impacts of COVID-19 on healthcare providers’ psychological health and well-being continue to affect their professional roles and activities, leading to compassion fatigue. The purpose of this review was to identify and summarize published literature on compassion fatigue among healthcare providers and its impact on patient care. Six databases were searched: MEDLINE (Ovid), PsycINFO (Ovid), Embase (Ovid), CINAHL, Scopus, Web of Science, for studies on compassion fatigue in healthcare providers, published in English from the peak of the pandemic in 2020 to 2023. To expand the search, reference lists of included studies were hand searched to locate additional relevant studies. The studies primarily focused on nurses, physicians, and other allied health professionals. This scoping review was registered on Open Science Framework (OSF), using the Preferred Reporting Items for Systematic reviews and Meta-Analysis (PRISMA) extension to scoping review. From 11,715 search results, 24 met the inclusion criteria. Findings are presented using four themes: prevalence of compassion fatigue; antecedents of compassion fatigue; consequences of compassion fatigue; and interventions to address compassion fatigue. The potential antecedents of compassion fatigue are grouped under individual-, organization-, and systems-level factors. Our findings suggest that healthcare providers differ in risk for developing compassion fatigue in a country-dependent manner. Interventions such as increasing available personnel helped to minimize the occurrence of compassion fatigue. This scoping review offers important insight on the common causes and potential risks for compassion fatigue among healthcare providers and identifies potential strategies to support healthcare providers’ psychological health and well-being.

**Supplementary Information:**

The online version contains supplementary material available at 10.1186/s12913-023-10356-3.

## Highlights

• What do we already know about this topic? The elevated and persistent mental stress associated with the COVID-19 pandemic predisposed healthcare providers (HCP) to various psychological conditions such as compassion fatigue. Declines in health providers’ mental health has been observed to negatively impact their professional performance and the quality of patient care.

• How does your research contribute to the field? This review provides an overview of the prevalence of compassion fatigue among HCPs across the globe during the COVID-19 pandemic. The main risk factors for compassion fatigue include younger age, female sex, being either a physician or a nurse, high workload, extensive work hours, and limited access to personal protective equipment (PPE). Negative behavioral intention towards patients has been identified to be a consequence of compassion fatigue. Interventions such as the provision of emotional support, increased monitoring for conditions such as stress and burnout, and increasing available personnel helped to minimize the occurrence of compassion fatigue.

• What are your research’s implications towards theory, practice, or policy? While the public health emergency associated with the COVID-19 pandemic has ended, the impact on human health resources persists. The findings of this review can inform policy decisions and implementation of evidence-based strategies to prevent, manage, and lessen the negative effects of compassion fatigue on HCPs and its subsequent impacts on patient care.

**Supplementary Information:**

The online version contains supplementary material available at 10.1186/s12913-023-10356-3.

## Introduction

The 2019-novel coronavirus disease (COVID-19) outbreak spread rapidly and by January 30^th^, 2022 was formally proclaimed a global health emergency despite being first identified just over a month prior [1]. Although there have been five other global health emergencies associated with disease outbreaks since 2009, none has matched the scale and scope of the COVID-19 pandemic [2]. In the short-term the rapid increase in patients requiring acute care services presented unprecedented challenges for health systems. Care provision and infection control strategies were hampered by capacity limitations, staffing shortfalls and supply chain challenges [3]. As a result, healthcare providers (HCPs) encountered mounting levels of strain which have continued with little reprieve for the duration of and beyond the global COVID-19 pandemic. Limited access to personal protective equipment (PPEs) exacerbated transmission of the virus, compounding healthcare providers’ fears of contracting and spreading COVID-19 among their peers, patients and families [4–7]. HCPs also contracted COVID-19, became seriously ill and died with global estimates of HCP death between January 2020 and May 2021 being over 100,000. With time, the number of absences, extended sick leaves and staff turnovers increased [7, 8]. The combination of short staffing, frequent changes to workflow and continuous care provision to patients who were gravely ill and had high mortality amplified the toll on health care providers [8, 9]. While no longer a global health emergency, there continue to be COVID-19 cases and deaths. As of July 14, 2023 there were 767,972,961 COVID-19 cases and 6,950,655 deaths globally [10].

HCPs around the globe who treated severe COVID-19 cases, a process which necessitated in-depth compassionate engagement, became vulnerable to developing compassion fatigue as a result of their continued and in-depth involvement in the care of these severely ill patients and their families [11]. Compassion fatigue is defined as a composite of two measurements: burnout (sustained employment-related stress that compromises an individual’s desire to work) and secondary trauma (the development of traumatic symptoms resulting from the protracted exposure to the suffering of others) [12, 13]. An individual experiencing compassion fatigue has a reduced ability for showing compassion to others, resulting from the prolonged exposure to witnessing the suffering of others without being able to relieve one’s anguish despite having the desire to do so [9]. Individuals experiencing compassion fatigue may express a range of behaviors such as increased work absences or declines in the ability to engage in work-related tasks such as decision-making. Burnout and secondary trauma are suggested to be mediated by compassion satisfaction—the pleasure that comes from helping behavior [11, 12].

As the pandemic shifts from being a global health emergency to an endemic disease, there continues to be concern for HCP health and well-being [14–16]. The increased and chronic nature of the stress experienced during and beyond the COVID-19 pandemic has heightened HCPs risk for a range of negative psychological impacts such as depression, fearfulness, grief and post-traumatic stress disorder (PTSD) [17]. Prior infectious disease outbreaks (SARS-CoV-1, H1N1, MERS-CoV, Ebola) are also associated with an increased prevalence of declining mental health in HCPs [18]. A growing body of research on the COVID-19 pandemic highlights the range of psychological symptoms HCPs developed following their sustained exposure to COVID-19 including burnout, feelings of isolation, insomnia, grief, emotional exhaustion, depression, post-traumatic stress and depersonalization, some of which have persisted over time [14, 17, 19–22]. The consequences of HCPs’ declining psychological health and well-being has had impacts on the quality of patient care and indirectly on patient outcomes through inadequate staffing [18]. Compromises in HCPs’ ability to provide optimal clinical care can have serious consequences, including the worsening of patient conditions and the increased transmission of the infection from patients to others in the hospital [18]. In addition, compassion fatigue may be exacerbated by the COVID-19 pandemic, potentially leading to moral injury, decreased productivity, increased turnover, and reduced quality of care [23]. Moreover, a growing body of literature suggests that challenges across health systems will persist although COVID-19 is no longer a global health emergency [24, 25]. As such, it is important to have a fulsome understanding of COVID-19’s toll on HCPs and tailor health system strategies accordingly.

As health care systems continue to experience a health human resources crisis, it is important to identify and understand the prevalence of compassion fatigue, identify contributing factors, and increase understanding of the consequences and actions that can be taken to address compassion fatigue among HCPs. While there has been in an increase in the body of published literature on the health and well-being of HCPs since the onset of the COVID-19 pandemic, there continues to be a knowledge gap mapping the incidence of compassion fatigue, its resultant impact on HCP well-being, and its potential influence on patient care provision [11, 17]. A comprehensive review of the literature on compassion fatigue among HCPs can inform policy and practice initiatives to improve the current health human resources crisis experienced by many health systems. It may also aid in identifying prospective research foci.

## Review aim

The purpose of this scoping review was to synthesize and provide a synopsis of the literature on compassion fatigue among HCPs during the COVID-19 pandemic and to understand its broader impact. The review was guided by the following question: What is the current state of knowledge on compassion fatigue among HCPs over the course of COVID-19?

## Methods

### Project registration

This scoping review was registered under Open Science Framework. A project outline was submitted including the study hypotheses, design, and data collection procedures. The DOI for the registered project is as follows: 10.17605/OSF.IO/F4T7N. In addition, a scoping review protocol for this review has been published in a peer-reviewed journal (10.1136/bmjopen-2022-069843).

### Study design

A systematic scoping review strategy was chosen to explore the existing body of literature pertaining to the research topic. The objective of a scoping review is to identify relevant literature on a given topic, without focusing on evaluating research quality or conducting a thorough analysis of selected studies, as systematic reviews typically do. Current gaps in research and directions for future research can be identified by means of summarizing emerging literature on compassion fatigue in HCPs.

The current scoping review used two methodological tools, namely the Arksey and O’Mally scoping review framework as well as the Joanna Briggs Institute Critical Appraisal Tools. The Arksey and O’Malley framework comprises five stages, which include: (1) formulating the research question; (2) identifying relevant studies; (3) selecting studies for inclusion; (4) extracting and organizing the data; and (5) collating, summarizing, and reporting the findings [26]. While scoping reviews typically do not require article appraisal, all articles were evaluated by one author (CO) using the methodology established by the Joanna Briggs Institute (JBI) to enhance the overall quality of the review [27]. No articles were excluded based on their quality, in accord with the Arksey and O’Malley framework [26].

### Stage I: Identifying the research question(s)

 The research objective and question were drafted by the authors (AG, LH, CO, SB) and can be found in the previous section under “Research aim”.

### Stage II: Identifying relevant studies

As outlined by the JBI methodology, a three-step approach was used to identify relevant studies. These steps include: (1) conducting a preliminary search of at least two suitable databases; (2) identifying relevant keywords and index terms to perform a secondary search across all chosen databases; and (3) manually examining the reference lists of the included articles to discover additional relevant studies [28] ^ (p11)^.

#### Preliminary literature search

To establish the criteria for inclusion and exclusion, an initial and restricted search was conducted on the subject of interest. The preliminary literature exploration encompassed three scholarly electronic databases: MEDLINE (Ovid), Scopus, and Web of Science. The search employed the keywords “compassion fatigue” and incorporated the timeframe March 1, 2020, to June 15, 2022, so that the most impactful waves of the COVID-19 pandemic were represented in the included literature, resulting in 1519, 2489, and 2246 studies, from the respective databases. These three databases were selected due to their likelihood of yielding results relevant to the research topic. To construct a comprehensive search strategy, a collection of keywords and index terms were identified from the titles and abstracts of relevant articles. The search strategy was further refined in collaboration with a social science librarian.

#### Structured search strategy

A systematic search was conducted across six scholarly electronic databases: MEDLINE (Ovid), Embase (Ovid), CINAHL, Scopus, and Web of Science. These databases were deliberately chosen to encompass a broad range of relevant findings within the current knowledge landscape regarding the research topic. The systematic search of the literature commenced once the scoping review was peer reviewed and revisions were addressed by the authors. Using the selected vocabulary and Boolean connectors as shown in Table 1, a string of relevant search terms was developed. The search strategy was adapted accordingly for each individual database (e.g., Medical Subject Headings [MeSH] terms for MEDLINE [Ovid]). In the final stage of the search strategy, the reference lists of all included studies were manually examined to identify additional relevant studies.

**Table 1:** Search strategy

Search strategy criteria	Search strategy
Databases	MEDLINE (Ovid), PsycINFO (Ovid), Embase (Ovid), CINAHL, Scopus, Web of Science
Language filter	English
Time filter	January 2020—May 2023
Geographic filter	None
Keywords	1. “coronavirus” OR “coronavirinae” OR “2019-nCoV” OR “2019nCoV” OR “nCoV2019” OR “nCoV-2019” OR “COVID-19” OR “COVID19” OR “SARS-CoV-2” OR “SARSCoV-2” OR “SARSCoV2” OR “SARS-CoV2” OR “SARSCov19” OR “SARS-Cov19” OR “SARS-Cov-19” OR “severe acute respiratory syndrome” OR “NcovWuhan” OR “NcovHubei” OR “NcovChina” OR “NcovChinese” 2. “compassion fatigue” OR “burnout” OR “secondary trauma” OR “secondary traumatic stress” OR “secondary traumatization” OR OR “vicarious trauma” OR “vicarious traumas” OR “vicarious traumatization” 3. “healthcare providers” OR “healthcare provider” OR “health personnel” OR “medical staffs” OR “medical staff” OR “hospital staff” OR “hospital staff” OR “health personnel” OR “nurse” OR “nurses” OR “physicians” OR “practitioners” OR “practitioner” OR “clinician” OR “clinicians” 4. “compassion satisfaction” 1. AND 2. AND 3. AND 4
Inclusion criteria	Population: healthcare providers Concept: compassion fatigue Context: formal healthcare settings
Exclusion criteria	Studies not meeting the above-stated PCC criteria Study designs: editorial, letter to the editor, review

#### Inclusion criteria

The inclusion criteria for this review was formulated using the PCC (Population, Concept, Context) mnemonic developed by JBI (Table 1). The participants included in this review were HCPs who were employed across healthcare systems during the COVID-19 pandemic (e.g., physicians, registered nurses, nurse practitioners, physician assistants, and licensed clinical social workers). The concept explored in this review focused on compassion fatigue among HCPs working in healthcare systems during the COVID-19 pandemic. The context of the study encompassed various care settings where HCPs carry out their professional activities across different clinical specialties (e.g., surgery, critical care, palliative care), as well as clinical settings (e.g., inpatient and outpatient). For the purposes of this scoping review, formal healthcare settings were broadly classified as those that provided health services and were situated within and administered by healthcare institutions.

This scoping review only included articles published in English. A time filter was applied to encompass studies conducted between 2020 to 2023, spanning the period from the onset of the COVID-19 pandemic to the present. A range of study designs were included in the review (i.e., experiments, quasi-experimental studies, analytical observational studies, descriptive observational studies, mixed-methods studies, and qualitative studies).

#### Exclusion criteria

Through the past two decades, compassion fatigue has been defined in different ways, sometimes being considered synonymous with burnout and secondary traumatic stress, or as an outcome resulting from both components [12, 13]. Yet recently, it has been suggested that compassion fatigue is a focal concept related to the management of traumatic situations whereas burnout is a general concept that may have multiple contributors [26]. Due to the conceptual ambiguity surrounding compassion fatigue, articles that solely examine the components of compassion fatigue, such as burnout and secondary trauma, without directly addressing compassion fatigue itself, were excluded from consideration.

Studies that failed to meet the inclusion criteria or lacked full-text availability were excluded from the review. Additionally, editorials, letters to the editor, commentaries, and reviews were also excluded as they did not offer sufficient information for addressing the research questions.

### Stage III: Study selection

After the full database searches were conducted, all identified citations were compiled and uploaded into Covidence. Any duplicate citations were automatically excluded.

Three reviewers (LH, CO, AG) independently screened the titles and abstracts of the identified studies to assess their eligibility according to the pre-established inclusion and exclusion criteria. Subsequently, the full texts of 736 selected studies were evaluated to arrive at the final list of articles for data extraction. The reasons for excluding specific studies were documented. Throughout the process, any disagreements that arose at each stage of study selection were resolved through discussions with a third reviewer (AG, SB).

**Fig. 1:**
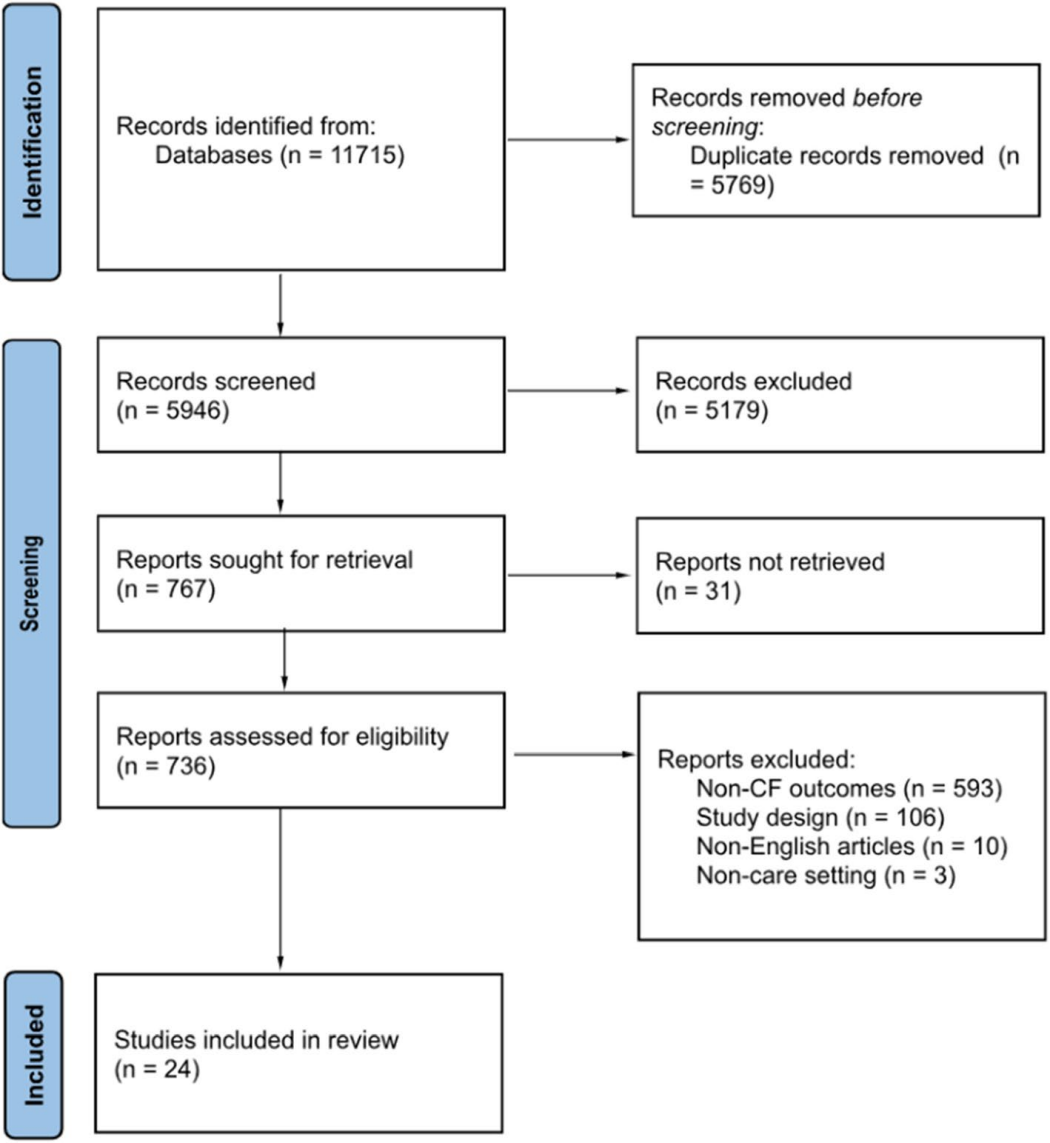
PRISMA flow chart [28]

The outcomes of the study selection process were presented in a flow diagram adhering to the Preferred Reporting Items for Systematic Reviews and Meta-Analyses extension for scoping reviews (PRISMA-ScR) guidelines (Fig. 1) [29]. Additionally, all the included studies underwent an assessment of their risk of bias (quality) using established critical appraisal tools from the Joanna Briggs Institute (JBI) for Evidence Synthesis [30]. Although not mandatory for scoping reviews, appraisals of study quality will contribute to the subsequent implications and future steps stemming from this scoping review [31]. The JBI provides critical appraisal checklists for various study designs, encompassing experimental, quasi-experimental, randomized controlled trials, observational, and qualitative study designs. One reviewer (CO) conducted the assessments of all the included studies, and a second reviewer (AG) verified the evaluations. Any discrepancies that arose were discussed and resolved in consultation with both reviewers. In line with the methodology of scoping reviews, no studies were excluded based on their quality assessments, ensuring a comprehensive understanding of the current state of the literature on compassion fatigue among HCPs during the COVID-19 pandemic. A summary of the quality assessments were presented in the results section of the review, while the full appraisals can be found in Additional file 1.

### Stage IV: Data extraction

To facilitate data extraction aligned with the research objectives, a data-extraction template was developed by one reviewer (LH). This template encompassed various aspects of the included studies (i.e., authors, publication year, study populations, country, study design, aims, sample size, assessment instruments, risk factors, protective factors, consequences of compassion fatigue, and measures to prevent/manage/reduce compassion fatigue). Utilizing Covidence, two independent reviewers (LH, CO) extracted the relevant data from the studies included in the final list of citations.

### Stage V: Risk of bias

Standardized tools developed by the Joanna Briggs Institute for respective study types were used to assess risk of bias (quality) for all studies included in the review [27]. The study appraisals were conducted by one reviewer (CO) and reviewed by another reviewer (AG). Any discrepancies were discussed and resolved together. While no studies were excluded based on the appraisal scores to ensure a comprehensive presentation of the available literature on compassion fatigue among healthcare providers, the findings for the risk of bias assessments are summarized in the results section and the full appraisals are presented in Additional file 1.

### Stage VI: Collating, summarizing, and reporting the results

To summarize and synthesize the findings, the study followed a three-step approach proposed by Levac et al. [32]: (1) collating and analyzing the collected data; (2) reporting the results and outcomes to address the study objectives; and (3) discussing the potential implications that findings hold for future research and policy considerations [31]. The review process adhered to the PRISMA Extension for Scoping Reviews checklist, which provided guidance for conducting the review and reporting the findings [26].

## Results

### Search results

Figure 1 displays the PRISMA-ScR flowchart of the scoping review search strategy. The search and reference list initially yielded 11,715 studies. Of these, 5769 were excluded as duplicates. Following the title and abstract screening of the remaining studies, 5179 studies were excluded as they met the exclusion criteria. Finally, the full-texts of the remaining 736 studies were screened, and 712 were excluded as they did not meet the inclusion criteria. In total, 24 eligible studies were included in the review for further analysis.

### Risk of bias of included studies

The complete assessment of risk of bias of all 24 included studies is available in Additional file 1. Within the two mixed-methods studies risk of bias primarily stemmed from the quantitative strand of the studies with a lack of clarity provided about study inclusion criteria, study setting, and identification of confounding factors [29]. Other sources of bias in other quantitative studies were vagueness around the criteria used for outcome measurement [30] and only one study identified potential cofounding factors along with strategies to manage them [31]. Further shortcomings related to the failure to provide transparency around the use of valid and reliable outcome measures [23, 31, 33–42]. Within qualitative studies not all provided information about the researchers’ theoretical stance [29, 41, 43] and two studies did not provide documentation of ethics approval for the conducted research [43, 44]. One included case report met most assessment criteria for risk of bias although more description of assessment, post-assessment condition and adverse events were warranted [45].

### Characteristics of studies

Study characteristics are presented in Table 2. Of the 24 eligible studies, 18 studies used quantitative methods [23, 30, 31, 33–40, 46–51], 3 studies used qualitative methods [43–45], and the remaining studies used mixed-methods approaches [29, 41, 52]. Additionally, 13 studies focused on the antecedents of compassion fatigue [23, 29, 33–36, 40–42, 45–48] and 5 studies examined the consequences of compassion fatigue [30, 37, 43, 44, 49]. Six studies were conducted in the United States, with the others being conducted in a range of countries including Ecuador, Spain, United Kingdom, Italy, Greece, Turkey, Iran, Uganda, Taiwan, Japan, Philippines, China, and India. These studies primarily focused on nurses, physicians, and other allied health professionals. The study samples included both male and female HCPs. Only one study focused exclusively on female HCPs [43].

**Table 2:** Characteristics of included studies on compassion fatigue in HCPs during the COVID-19 pandemic

Author, year	Study design	Study period	Participants	Country
Austin, 2021 [35]	Qualitative	March–July 2020	15 HCPs	United States
Carmassi, 2022 [40]	Cross-sectional	July 2018–January 2019	126 HCPs	Italy
Cheng, 2021 [23]	Cross-sectional	November–December 2020	598 Nurses	China
Cuartero-Castaner, 2021 [41]	Cross-sectional	April–July 2020	117 HCPs	Ecuador
Hochwarter, 2022 [36]	Longitudinal	October–November 2020	175 Nurses	United States
Kase, 2022 [37]	Cross-sectional	June–July 2020	499 Physicians	United States
Kaya, 2022 [24]	Cross-sectional	March 2021	407 Nurses	Turkey
Kong, 2022 [38]	Qualitative	March–April 2020	20 Physicians	United Kingdom
Kottoor, 2022 [25]	Cross-sectional	2020	107 Nurses	India
Labrague, 2021 [39]	Cross-sectional	November–December 2020	270 Nurses	Philippines
Moreno-Mulet, 2021 [26]	Mixed-methods	June–November 2020	122 HCPs	Spain
Nishihara, 2022 [27]	Case study	–	–	Japan
Perez-Chacon, 2021 [28]	Cross-sectional	April–May 2020	694 HCPs	Spanish-speaking countries
Ramaci, 2020 [29]	Cross-sectional	March 2020	260 HCPs	Italy
Ruiz-Fernandez, 2020 [30]	Cross-sectional	March–April 2020	506 HCPs	Spain
Ruiz-Fernandez, 2021 [42]	Cross-sectional	March–April 2020	506 HCPs	Spain
Stevenson, 2021 [31]	Cross-sectional	May 2020	224 Nurses	United States
Su, 2021 [43]	Cross-sectional	July–April 2020	503 HCPs	Taiwan
Zakeri, 2022 [33]	Cross-sectional	April 2019–July 2020	508 Nurses	Iran
Missouridou, 2021 [34]	Mixed-methods	May–October 2020	105 Nurses	Greece
Amir, 2022 [13]	Cross-sectional	April 2020	395 Nurses	Uganda
Yilmaz, 2022 [48]	Cross-sectional	June 2020	796 HCPs	Turkey
Spiridigliozzi, 2022 [49]	Cross-sectional	2021	98 HCPs	United States
Gribben, 2023 [53]	Mixed-methods	June 2020–October 2021	499 Physicians	United States

A variety of assessment tools were used to measure compassion fatigue across included studies. Common tools included Compassion Fatigue Short Scale (CFSS) [33, 47, 48], Compassion Fatigue Scale (CFS) [30, 49], Professional Quality of Life Scale Version 5 (ProQoL 5) [23, 29, 29, 31, 35, 36, 38–42, 50, 51], Work-Related Quality of Life Scale (WRQoL) [46], and Compassion Fatigue and Satisfaction Self-Test (CFST) [37, 52] (Table 3).

**Table 3:** Main results of reviewed studies

Author, year	Tools	Scores	Antecedents	Consequences
Austin, 2021 [35]	–	–	Inability to cope with rapid change Decreased social interactions	–
Carmassi, 2022 [40]	ProQOL 5	11.3 ± 4.2	Psychiatric comorbidities	–
Cheng, 2021 [23]	CFSS	48.79 ± 25.95	Psychiatric comorbidities	Negative behavioural intentions towards patients
Cuartero-Castaner, 2021 [41]	ProQOL 5	24.84 ± 7.4	Decrease in hardiness Working with emotionally tolling cases	–
Hochwarter, 2022 [36]	CFS	3.25 ± 1.63	Psychiatric comorbidities Inability to cope with rapid change	–
Kase, 2022 [37]	CFST	18.32 ± 0.6	–	–
Kaya, 2022 [24]	WRQoL	1.67 ± 0.90	Less work experience Working with emotionally tolling cases	Lower job satisfaction Reduced professional commitment
Kong, 2022 [38]	–	–	Inability to cope with rapid change Fear of infection Increased workload Increased working hours	–
Kottoor, 2022 [25]	CFSS	33.63 ± 22.59	Fear of infection	–
Labrague, 2021 [39]	CFS	2.213 ± 0.979	–	Lower job satisfaction Reduced professional commitment Negative behavioural intentions towards patients
Moreno-Mulet, 2021 [26]	ProQOL 5	26.5 ± 6.2	Inability to cope with rapid change Fear of infection Increased workload Increased working hours Lack of access to PPEs	–
Nishihara, 2022 [27]	–	–	Increased workload Increased working hours	–
Perez-Chacon, 2021 [28]	ProQOL 5	21.16 ± 7.95	Excessive empathetic engagement, sensitive sensory processes, and overidentification	–
Ramaci, 2020 [29]	ProQOL 5	–	Increased workload Stigma	–
Ruiz-Fernandez, 2020 [30]	ProQOL 5	20.5 ± 7.95	Psychiatric comorbidities Working with emotionally tolling cases	–
Ruiz-Fernandez, 2021 [42]	ProQOL 5	19.86 ± 7.62	Excessive empathetic engagement, sensitive sensory processes, and overidentification	–
Stevenson, 2021 [31]	CFSS	–	Working with emotionally tolling cases	–
Su, 2021 [43]	ProQOL 5	20.9 ± 7.6	Psychiatric comorbidities	–
Zakeri, 2022 [33]	ProQOL 5	49.70 ± 11.35	–	–
Missouridou, 2021 [34]	ProQOL 5	22.46 ± 6.76	Inability to cope with rapid change Psychiatric comorbidities	Feelings of guilt, powerlessness, and frustration
Amir, 2022 [13]	ProQOL 5	–	Increased workload Increased working hours Working with emotionally tolling cases	–
Yilmaz, 2022 [48]	ProQOL 5	16.09 ± 8.27	Fear of infection	–
Spiridigliozzi, 2022 [49]	ProQOL 5	–	Inability to cope with rapid change	–
Gribben, 2023 [53]	CFST	18.6 ± 0.5	Working with emotionally tolling cases Fear of infection Inability to cope with rapid change Social isolation	–

*Tools*: *CFSS *Compassion Fatigue Short Scale ([range: 13–130], *CFS* Compassion Fatigue Scale [low: <  = 30; moderate: 31–35; high: >  = 36], *ProQoL 5* Professional Quality of Life Scale Version 5 ([low: =  < 22; moderate: 23–41; high: >  = 42], *WRQoL* Work-Related Quality of Life Scale [low: =  < 74; moderate: 75–81; high: >  = 82], and *CFST* Compassion Fatigue and Satisfaction Self-Test [low: <  = 30; moderate: 31–35; high: >  = 36]

**Fig. 2:**
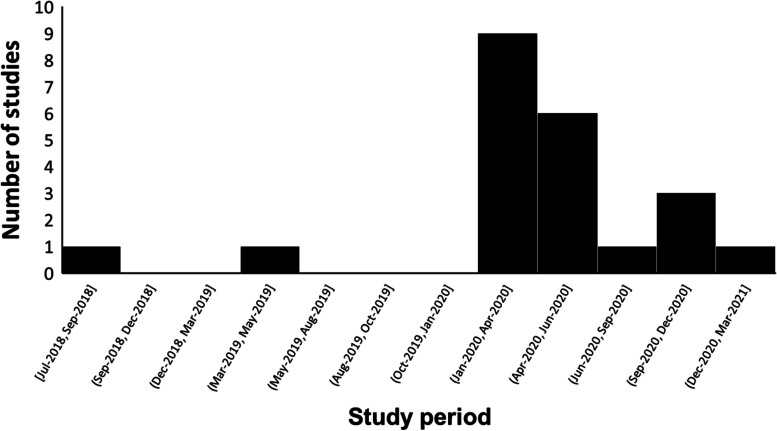
The time trend of study periods on compassion fatigue in HCPs during the COVID-19 pandemic

The time period of the study period shows that most of the studies were conducted in the first six months of 2020, coinciding with the World Health Organization’s declaration of the COVID-19 outbreak as a pandemic [54]. No studies included in the review were conducted between March 2021 and May 2023 (Fig. 2).

Findings were synthesized and presented using the following 4 themes: (1) prevalence of compassion fatigue, (2) antecedents of compassion fatigue (individual-Level, organizational-Level, and systems-level factors), (3) consequences of compassion fatigue, and (4) interventions for compassion fatigue.

### Theme 1: Prevalence of compassion fatigue

Of the studies reviewed, five measured the prevalence of compassion fatigue among HCPs during the COVID-19 pandemic [23, 30, 31, 36, 41]. In a study conducted in Spain, 306 out of 506 (60.4%) HCPs reported high levels of compassion fatigue while 170 (33.6%) showed moderate levels of compassion fatigue (ProQoL 5: M = 19.9, SD = 7.6) [36]. In a sample composed of 395 Ugandan frontline nurses, 49.11% of the nurses reported high levels of compassion fatigue, while 29.6% experienced moderate levels of compassion fatigue [23]. Over half of the nurses in the study (54.94%) reported direct exposure to COVID-19 cases. A study conducted in Greece found that in a sample of 105 nurses, the majority of nurses (51.4%) experienced moderate levels of compassion fatigue (ProQoL 5: M = 22.26, SD = 6.76) [41]. In a Taiwanese study of 503 HCPs, the majority of the participants (63.2%) experienced low levels of compassion fatigue (ProQoL 5: M = 20.9, SD = 7.6) [31]. Finally, in a Filipino sample composed of 270 frontline nurses, 61.4% of the nurses reported low levels of compassion fatigue (CFS: M = 2.213, SD = 0.979) [30].

### Theme 2: Antecedents of compassion fatigue

#### Individual-level factors

Age and sex were key factors associated with compassion fatigue among participant HCPs. Younger HCPs with less experience were more likely to experience mental health issues and conflicting feelings with regards to providing care to COVID-19 patients [23, 29, 44, 46]. Seven studies included in the review determined that female HCPs were more likely than male HCPs to experience compassion fatigue [23, 35, 36, 38, 40, 50, 52]. Physicians were also reported to have higher levels of compassion fatigue compared to nurses in three studies [36, 38, 39]. While nursing assistants had higher levels of compassion fatigue when compared to nurses in one study (ProQol 5: Nursing assistants = 29.15 ± 6.94; Nurse = 25.68 ± 5.87) [29]. Furthermore, the risk was higher in permanent workers compared to temporary workers (ProQoL 5: Permanent = 2.48 ± 1.29; Temporary = 2.11 ± 1.15; *P*-value < 0.05) [35]. One included study determined that marital status and education levels were not correlated with compassion fatigue [23]. Psychiatric comorbidities such as past trauma, burnout, stress, anxiety, and depression exacerbated HCPs’ psychological well-being across a number of included studies [31, 33, 36, 38, 39, 41, 49, 50]. Other psychological factors such as excessive empathetic engagement, sensitive sensory processes, and overidentification from frequent witnessing of patient suffering and deaths were found to aggravate the development of compassion fatigue [34, 39, 45]. The inability to cope with the rapidly evolving landscape of healthcare provision and a lack of self-care contributed to increased burden and blurring of role boundaries between professional and private lives [29, 41, 43, 44, 51, 52]. One study that used Compassion Fatigue and Satisfaction Self-Tests and a questionnaire of personal and professional characteristics found that feelings of underappreciation, insufficient compensations, and social isolation incurred psychological burden on pediatric sub-specialists [52]. Additionally, a decrease in occupational hardiness, as measured by the Occupational Hardiness Questionnaire, increased the risk of compassion fatigue among HCPs in two studies [42, 50]. Negative outcomes to the HCPs’ families and concerns revolving around their patients’ families also predicted higher risk of experiencing compassion fatigue [45, 48, 52]. Finally, HCPs’ fear of COVID-19 with regards to infection and transmission was identified as a predictor of compassion fatigue [29, 40, 43, 44, 47].

Two studies identified social support from family, friends, peers, and hospital leadership as a crucial protective factor for compassion fatigue [43, 52]. Coping mechanisms such as venting and exercising were found to help alleviate stress among HCPs [44]. Psychological qualities such as compassion satisfaction, professional satisfaction, resilience, vigor, and hardiness were found to help protect the psychological health of HCPs as well as reducing turnover intention and increasing perceived quality of care [30, 34, 36, 37, 39, 40, 42, 46, 50]. Self-care, self-awareness of limitations, and self-regulation of emotions were crucial for reducing risk of compassion fatigue in two studies comprised of physicians and nurses [44, 50]. Lastly, spirituality, religiosity, and meditation also served as protective factors in three studies on compassion fatigue in HCPs [41, 44, 51].

#### Organizational-level factors

In five of the articles reviewed, increased workload [23, 29, 44, 45], long working hours [23, 29, 44, 45], and increased number of patients [50] were identified as common predictors of compassion fatigue. Furthermore, providing direct care to COVID-19 patients, which were often emotionally challenging cases, exacerbated the psychological risks to HCPs [23, 36, 46, 48, 50]. Chronic exposure to a dynamic work environment also increased the risk of compassion fatigue among HCPs [29]. Lack of access to suitable PPEs and lack of foresight from management and human resources teams regarding infection control guidelines contributed to HCPs’ distress [29]. Adjusting to the discomfort caused by wearing PPEs presented as a challenge to maintaining the efficiency of work activities [29]. Lastly, in two studies, HCPs identified that while there were plenty of wellness resources provided by healthcare organizations to support mindfulness, there was a lack of practical and pragmatic resources for social and emotional support, work-life balance, and remuneration [23, 43].

Positive work conditions, such as a visible presence and engagement by leadership and management, as well as a positive work culture allowing HCPs to seek help without fear of judgment was found to be important protective factors against the development of compassion fatigue [44]. The social aspects of teamwork facilitated the sharing of feelings of trauma which in turn contributed to resilience and improved psychological well-being among HCPs in three studies [41, 43, 44]. One study observed that workplace wellness activities and a sense of feeling valued can prevent high levels of compassion fatigue [52]. Words of appreciation from supervisors boosted morale for some HCPs [44]. Attention to workplace safety in the form of PPEs and early access to vaccines alleviated the fear of infection [44]. Finally, two studies determined that adequate preparation and education to handle COVID-19 cases and increased autonomy decreased the risk of compassion fatigue and increased professional fulfillment [42, 44].

#### Systems-level factors

Significant and frequently changing public health measures over the course of the pandemic presented a challenge as they were disruptive to workflow and resulted in uncertainty, feelings of inadequacy, and distress among HCPs across a range of geographical contexts [29, 41, 43, 49]. Increases in the incidence of COVID-19 cases also contributed to a rise in the number of hospital admissions, aggravating HCPs’ workload [35]. Social-distancing policies precluded informal team interactions, such as sharing meals together, which posed a risk to HCPs’ psychological well-being by decreasing social support [43, 52]. Transitions to tele-health also increased social isolation [43]. A theme that emerged was the negative impact of stigma on HCPs, with their proximity to contagion, as a possible risk factor [35, 41]. Aggressive behaviors and verbal abuse from patients were sources of emotional stress for some HCPs [44]. Finally, negative peer pressure was identified as a barrier to HCPs engaging in self-care as they felt pressure to conform to sociocultural norms of an expected level of dedication [44]. In contrast to the impacts of stigma, a positive perception of one’s own profession is related to increased commitment and decreased compassion fatigue [46].

### Theme 3: Consequences of compassion fatigue

The findings of one study suggested that compassion fatigue associated with HCP’s professional practice impacted their private lives, predicting greater parental burnout (*r* = 0.542), child abuse (*r* = 0.468), child neglect (*r* = 0.493), spouse conflict (*r* = 0.340), and substance abuse (*r* = 0.298) [48]. This study identified factors such as direct care of COVID-19 patients (*r* = 0.255), exposure to patient death and suffering due to COVID-19 (*r* = 0.281), and family income loss due to COVID-19 (*r* = 0.366) as risk factors for compassion fatigue [48]. Additionally, at an organizational-level, two studies conducted in 2020 and 2021 observed that Turkish and Filipino HCPs who reported compassion fatigue also reported lower job satisfaction and reduced professional commitment [30, 46]. Consequently, elevated compassion fatigue also increased organizational turnover intent among Filipino HCPs (β = 0.301, *P*-value = 0.001) [30]. A study conducted in China found that compassion fatigue predicted negative behavioral intentions towards treating COVID-19 patients, as measured by the Attitude, Subjective Norms, and Behavioral Intention of Nurses toward Mechanically Ventilated Patients (ASIMP) questionnaire [33]. This suggests that quality of care may be adversely impacted [33]. Finally, an American study observed that compassion fatigue among HCPs was associated with deteriorating workplace culture [52].

#### Patient care

The provision of care during the pandemic was impacted by the general lack of preparation for handling novel tasks experienced by many HCPs [23]. Findings from one study found that many HCPs (73%) experienced a shift in their clinical practice setting, for example, from in-personal care to virtual telehealth consults as a result of the pandemic [43]. HCPs also experienced an increase in the need to provide palliative care as a result of the negative health impacts of COVID-19, something they may have had limited prior experience with [43]. In a case study conducted in Japan, the physician reported feeling inexperienced with handling the psychological impact of the pandemic experienced by not only the patients but also the patients’ family [45]. The consequences of not being able to provide optimal care was found to exacerbate feelings of guilt, powerlessness, and frustration in HCPs [41, 43]. In turn, study findings suggest that worsening compassion fatigue may reduce the quality of care provided by HCPs because it has been found to be a significant predictor of negative behavioral intention [30, 33, 40, 52].

### Theme 4: Interventions for compassion fatigue

Two studies in Japan and Uganda investigated potential interventions to support HCPs experiencing COVID-19 related compassion fatigue. On an individual-level, regularly engaging in self-care activities such as expressions of gratitude as well as learning how to recognize signs and symptoms of compassion fatigue were identified as crucial first steps in its management [45, 52]. Emotional support from colleagues and mental health specialists was found to be effective in improving the mental health of a Japanese physician experiencing compassion fatigue [45]. Findings of two studies identified the need for a systematic approach to monitor the progression of psychological symptoms and providing tailored resources in a timely manner to HCPs to help ameliorate compassion fatigue and its consequences [29, 45]. Suggested strategies included: facilitating regular consultations with each department [45, 52], increasing the staffing number of HCPs in busy departments [23, 45], and providing PPEs and vaccines in a timely manner [23, 52]. Lastly, findings from two studies in Uganda and the United States suggested that increased remuneration may prevent or minimize compassion fatigue [23, 52].

## Discussion

### Key findings

#### Characteristics of studies

This scoping review sought to provide a comprehensive summary of the literature published between January 2020 and May 2023 on the impact of the COVID-19 pandemic on compassion fatigue among HCPs and its subsequent impact on patient care. Most of the included studies were conducted in 2020 and used cross-sectional study designs. Given that the COVID-19 outbreak was declared a global health emergency in early 2020 [1], cross-sectional study designs were well-placed to provide prompt and important insights on compassion fatigue across the HCP population. Review findings were presented using four themes addressing the prevalence, antecedents, consequences, and consequences of compassion fatigue in HCPs. The prevalence of compassion fatigue was observed to vary across countries. The negative psychological outcomes reported by included studies were precipitated by individual-level factors such as age and occupational role; organizational-factors such as lack of access to PPE; and systems-level factors such as loss of social engagement and stigma. The consequences of compassion fatigue impacted HCPs’ personal and professional roles. Findings suggest an urgent need for policy makers, health managers, and team leaders to develop and implement strategies that target the potential root causes of compassion fatigue in HCPs.

#### Prevalence of compassion fatigue

Among the five studies that measured prevalence of compassion fatigue, results were highly variable across countries [23, 30, 31, 36, 41]. This may be attributed to differences in preparedness for infection containment and variability among health systems’ preparation and ability to respond to supply chain issues [53]. Taiwan provides an example of how digital technologies were adopted to improve disease surveillance and monitor medical supply chains [55]. Using the stringent Identify-Isolate-Inform model in conjunction with public mask-wearing and physical distancing, the spread of the disease was effectively contained in Taiwan [53]. Consequently, despite not enforcing lockdowns, Taiwan blocked the first wave of cases and slowed down subsequent outbreaks, which may contribute to the observed low prevalence of compassion fatigue among HCPs [56]. In the Philippines, responses to disease outbreaks varied across different municipalities and provinces [57]. Effective containment measures such as strict border control and early lockdowns in addition to plentiful medical supplies and personnel allowed certain regions to mount a strong response to this public health emergency, subsequently resulting in the observed low prevalence of compassion fatigue among HCPs [57]. In Uganda, there were generally low levels of preparedness with regards to the infection identification, PPE supply, access to hand-washing facilities, and establishment of isolation facilities [58]. This may have contributed to an overwhelmed healthcare system and overworked HCPs as the surge of cases was exacerbated by the shortage of disease containment resources [58]. In April 2020, Spain experienced the second highest infection incidence in the world [59]. The Spanish health system was overwhelmed by the abundance of patients due to lack of HCPs [60], hospital capacity, and material supplies [59]. An increase in compassion fatigue among HCPs was also observed in recent studies from Italy and Canada [61, 62]. Overall, the various strategies used to address the resultant COVID-19-related public health crisis presented distinctive challenges to HCPs in different countries. Caution must be taken when interpreting the study findings given the contextual differences across various healthcare systems. The psychological burden and prevalence of compassion fatigue subsequently varied depending on the context.

#### Antecedents of compassion fatigue

The findings of this review suggest that individual characteristics such as age and occupational role are significant contributing factors to the development of compassion fatigue during COVID-19 [63]. Specifically, older HCPs were less likely to experience compassion fatigue than younger HCPs according to regression analyses [23, 29, 44, 46]. This observation may be attributed to their increased work experience. Resilience was also positively linearly related to age [64]. Factors identified as potential contributors to the observed age-related advantage in wellbeing were access to job resources, better job security, work-life balance, and coping skills [64]. The compounding of stressors such as an increase in workload during the COVID-19 pandemic could have exacerbated the psychological health of younger HCPs. In the context of telework, older employees tended to create clear boundaries between work and non-work responsibilities [64]. The rise in telework among HCPs was mostly a consequence of the COVID-19 pandemic which may have increased the psychological burden on younger HCPs [65]. In addition, a study examining demographic predictors of resilience in nurses reported that younger nurses had less exposure to stress, and thus have fewer opportunities to develop skills in stress management [66]. As a result of these factors, the younger HCPs were at high risk for compassion fatigue during the COVID-19 pandemic. Interestingly, three of the included studies in this review also observed that physicians were at a higher risk of compassion fatigue compared to nurses [36, 38, 39]. This difference may be attributed to the burden of responsibility in relation to breaking bad news, a task that is often the physicians’ responsibility [67]. A study examining compassion fatigue in HCPs determined that conflict arising during patient interactions placed HCPs at a risk for compassion fatigue [68]. Delivery of bad or uncertain news also predicted a greater mental health burden in HCPs [68].

At the organizational level, findings from the studies included in this review identified that a lack of access to PPE was a contributor to compassion fatigue in HCPs during COVID-19 [29, 52]. Specifically, one study reported that the fear of infection and transmission to patients, family, and friends added to the concern of HCPs working in high-risk environments [69]. This finding can potentially be explained by the increased vulnerability that HCPs experience following a lag in the provision of PPE. Several organizational factors were determined as potential barriers to the distribution of PPE; the unprecedented nature of the pandemic presented challenges for maintaining domestic inventories [70]. Disruptions to the PPE global supply chain also amplified the equipment shortage [70]. This finding highlights the importance of monitoring and ensuring that domestic health supplies are adequately stocked.

At the system level, loss of social engagement [43, 52] and stigma [35, 41] were identified in the studies included in the review as antecedents to compassion fatigue. Public policies such as social-distancing and occupancy capacity limits negatively impact social interactions which may explain the loss of social engagement in addition to worsening mental health well-being in HCPs [71]. As certain practices transition to telehealth, other studies have found increased mental fatigue and difficulty with maintaining empathetic rapport, which has important implications on patient care [72, 73]. In addition, other studies have found that given the proximity of their role to contagion, stigma towards HCPs from patients increased during COVID-19 [74, 75]. Consequently, the combinatorial experience of being socially isolated and stigmatized may worsen mental health outcomes [76]. This points to a need for increased access to support services for HCPs such as virtual communities.

#### Consequences of compassion fatigue

Review findings suggest that compassion fatigue impacted the private and professional lives of HCPs. The risk for parental burnout has increased across many occupations during the pandemic [77]. Factors related to low levels of social support, lack of leisure time, and greater parental responsibilities in face of education disruptions adds to the psychological burden of parents [77]. HCPs were placed in a unique position having to work in highly stressful environments while also balancing household responsibilities and increased challenges related to childcare [48, 78]. This finding highlights a need for the provision of child support services for HCPs or a reduction in workload to alleviate the burden of parental and homecare responsibilities particularly in times of public health crises.

Beyond their private lives, this review has found that decreases in HCPs’ professional commitment due to compassion fatigue, may endanger the quality of patient care delivered [79]. In particular, this may be attributed to the surge in palliative care cases during the pandemic in conjunction with an unprepared workforce, creating psychological stress for HCPs [80]. In a study examining palliative care preparedness during the pandemic, a lack of core palliative care training and expertise among frontline HCPs [81] meant many felt emotionally unprepared to address cases with seriously ill patients [45]. An increased frequency of breaking bad news to patients’ families was associated with negative psychological outcomes [82]. Providing training on relevant communication skills may protect HCPs from compassion fatigue [83, 84].

#### Implications

The findings of this review highlight the urgency to provide support for HCPs who may be at risk for compassion fatigue which could have subsequent impacts on the provision of patient care [85]. To address the antecedents of compassion fatigue, this scoping review has identified a need for increased staffing, recruitment, and retention efforts on the part of hospital human resources departments [23, 45]. Interventions suggested by studies included in the review encompass the monitoring of psychological well-being among HCPs to inform timely provision of resources [29, 45]. Specifically, structured debriefing, training on self-care routine, reduced workload, and normalization of trauma-related therapy are essential interventions [86]. Additionally, a study identified that fostering collaborative workplace culture encourages social and emotional support among staff [45]. Certain hospitals have adopted “wobble rooms” as a private unwinding and venting space for employees [87]. Studies have observed that interventions aimed at improving the well-being of HCPs resulted in enhanced quality and safety of care being delivered [75].

### Strengths and limitations

There are both strengths and limitations in this review. Although some literature reviews focused on the psychological health status of HCPs (e.g., burnout, anxiety, depression), very few studies have specifically explored compassion fatigue. Reviews that considered the impact of the COVID-19 pandemic on HCPs were even more limited. It is known that compassion is a cornerstone of quality health care improvement and increases successful medical outcomes [88–90]. Nevertheless, prolonged exposure to distressing events by HCPs, such as patient death and suffering, results in the absorption of negative emotional responses and leads to the development of compassion fatigue [91]. This scoping review presents an extensive exploration of the current body of literature on compassion fatigue among HCPs during the COVID-19 pandemic. Another strength in this study lies in the transparency and reproducibility of the methodology. The scoping review protocol has been published in a peer-reviewed journal to establish high methodological standards for the final scoping review [92]. Additionally, the study plan was pre-registered with Open Science Framework to ensure commitment to the methodology. Double extraction was performed to ensure that a comprehensive descriptive summary of the studies was achieved.

Some limitations include the short time frame chosen for the included studies that were published since the COVID-19, which may have constrained the breadth and quality of the studies. Longitudinal studies may not be captured in the review as this study methodology requires a prolonged period of time to yield meaningful observations. More data is needed to support conclusions on the impact of compassion fatigue on patient care. Additionally, none of the studies included in the review were conducted between March 2021 and May 2023, which may miss out on meaningful trends in levels of compassion fatigue in HCPs. This scoping review only included literature published in English so studies published in other languages were not assessed. Additionally, no comparisons of compassion fatigue were made among the HCP groups in spite of potentially relevant differences such as patient exposure. There was also a lack of allied health profession representation, with the majority of the study population being nurses or physicians. Lastly, grey literature was not included in this scoping review which may delimitate the information included in the scoping review.

There were recurring themes related to limitations in the included research studies. Several studies identified sampling issues including small sample sizes, restricted sample frame, low response rate, and selection error [23, 29, 31, 38–43, 47, 50, 51, 83]. Other studies have called for investigations into how different sociodemographic factors, other psychiatric diseases, health care settings, and workplace environment impact compassion fatigue in HCPs [38, 39, 47, 48, 83]. One study observed a lack of homogeneity in the sample due to an overrepresentation of female HCPs in the sample [38]. Lastly, many studies employed a cross-sectional study design which limits the interpretation of the data in terms of causality [23, 30, 31, 34, 42, 47, 48, 50]. While there are limitations to the study, a comprehensive summary of existing literature may be useful to inform future research and policies.

Future research is needed to examine the longitudinal impacts of COVID-19 on compassion fatigue in HCPs. Moreover, research in this area could be strengthened by including a consultation phase with external experts on compassion fatigue to improve the robustness of the scoping review.

## Conclusions

The COVID-19 pandemic presented a unique set of challenges to healthcare systems across the globe. This scoping review indicated that the prevalence of compassion fatigue was inconsistent across countries and may reflect the variability of pandemic preparedness among the individual countries. Primary risk factors for the development of compassion fatigue included being younger, female, a physician or nurse, and having limited access to PPE in conjunction with an excessive workload and prolonged work hours. The negative impacts of compassion fatigue were experienced at the individual and organizational level. The findings suggest there is a systemic need to assess, monitor and support health professionals’ well-being particularly during conditions of protracted health crises such as a pandemic. In addition, many health systems and sectors are facing a profound health human resources crisis and therefore ongoing efforts must be made to improve workplace environments and increase recruitment and retention efforts. Lastly, pandemic planning must include provisions to support health providers’ ability to safely do their jobs while also minimizing negative impacts to their health and well-being.

### Supplementary Information

**Additional file 1.** Critical appraisals of included articles.

## Data Availability

All the material presented in the manuscript is owned by the authors and/or no permissions are required.
