# Supplementary material for: Compassion fatigue in healthcare providers: a scoping review
Source: BMC Health Serv Res. 2023 Dec 1;23:1336. doi: 10.1186/s12913-023-10356-3 (PMC10693134; doi:10.1186/s12913-023-10356-3)
Supplement: Supplementary file 1 — Additional file 1. Critical appraisals of included articles. [file 12913_2023_10356_MOESM1_ESM.docx]

**Critical Appraisals of Included Articles**

| Author(s), year | Reference | Joanna Briggs Institute Checklist Questions for Analytical Cross-Sectional Studies | | | | | | | | Score |
| --- | --- | --- | --- | --- | --- | --- | --- | --- | --- | --- |
|  |  | Were the criteria for inclusion in the sample clearly defined? | Were the study subjects and the setting described in detail? | Was the exposure measured in a valid and reliable way? | Were objective, standard criteria used for measurement of the condition? | Were confounding factors identified? | Were strategies to deal with confounding factors stated? | Were the outcomes measured in a valid and reliable way? | Was appropriate statistical analysis used? |  |
| Amir & Okalo, 2022 | [1] | NO | YES | NO | YES | NO | NO | NO | YES | 3/8 |
| Carmassi et al., 2022 | [2] | YES | YES | YES | UNSURE | NO | NO | NO | YES | 4/8 |
| Cheng et al., 2021 | [3] | YES | YES | YES | YES | NO | NO | NO | YES | 5/8 |
| Cuartero-Castaner et al., 2021 | [4] | NO | NO | YES | YES | NO | NO | YES | YES | 4/8 |
| Hochwarter et al., 2022 | [5] | UNSURE | NO | YES | YES | NO | NO | YES | YES | 4/8 |
| Kase et al., 2022 | [6] | YES | NO | NO | YES | NO | NO | NO | YES | 3/8 |
| Kaya et al., 2022 | [7] | YES | NO | YES | YES | NO | NO | YES | YES | 5/8 |
| Kottoor & Chacko, 2022 | [8] | YES | NO | YES | NO | NO | NO | YES | YES | 4/8 |
| Labrague & de los Santos, 2021 | [9] | YES | YES | YES | UNSURE | NO | NO | YES | YES | 5/8 |
| Missouridou et al., 2021 | [10] | NO | NO | NO | UNSURE | NO | NO | NO | YES | 1/8 |
| Moreno-Mulet et al., 2021 | [11] | NO | NO | YES | YES | NO | NO | YES | YES | 4/8 |
| Perez-Chacon et al., 2021 | [12] | YES | NO | UNSURE | YES | NO | NO | UNSURE | YES | 3/8 |
| Ramaci et al., 2020 | [13] | NO | NO | NO | YES | NO | NO | NO | YES | 2/8 |
| Ruiz-Fernandez et al., 2020 | [14] | YES | NO | NO | YES | NO | NO | NO | YES | 3/8 |
| Ruiz-Fernandez et al., 2021 | [15] | YES | YES | YES | YES | NO | NO | NO | YES | 5/8 |
| Spiridigliozzi, 2022 | [16] | UNSURE | NO | YES | YES | NO | NO | YES | YES | 4/8 |
| Stevenson et al., 2021 | [17] | YES | YES | YES | YES | NO | NO | YES | YES | 6/8 |
| Su et al., 2021 | [18] | UNSURE | NO | YES | YES | YES | YES | NO | YES | 5/8 |
| Yilmaz et al., 2022 | [19] | YES | YES | YES | YES | NO | NO | UNSURE | YES | 5/8 |
| Zakeri et al., 2021 | [20 | YES | NO | NO | YES | NO | NO | NO | YES | 3/8 |

| Author(s), year | Reference | Joanna Briggs Institute Checklist Questions for Case Reports | | | | | | | | Score |
| --- | --- | --- | --- | --- | --- | --- | --- | --- | --- | --- |
|  |  | Were patients’ demographic characteristics clearly described? | Was the patients’ history clearly described and presented as a timeline? | Was the current clinical condition of the patient on presentation clearly described? | Were diagnostic tests or assessment methods and the results clearly described? | Was the intervention(s) or treatment procedure(s) clearly described? | Was the post-intervention clinical condition clearly described? | Were adverse events (harms) or unanticipated events identified and described? | Does the case report provide takeaway lessons? |  |
| Nishihara et al., 2022 | [21] | YES | YES | YES | NO | YES | NO | NO | YES | 5/8 |

| Author(s),  year | Reference | Joanna Briggs Institute Checklist Questions for Qualitative Research | | | | | | | | | | Score |
| --- | --- | --- | --- | --- | --- | --- | --- | --- | --- | --- | --- | --- |
|  |  | Is there congruity between the stated philosophical perspective and the research methodology? | Is there congruity between the research methodology and the research question or objectives? | Is there congruity between the research methodology and the methods used to collect data? | Is there congruity between the research methodology and the representation and analysis of data? | Is there congruity, between the research methodology and the interpretation of results? | Is there a statement locating the researcher culturally or theoretically? | Is the influence of the researcher on the research, and vice-versa, addressed? | Are participants, and their voices, adequately represented? | Is the research ethical according to current criteria or, for recent studies, and is there evidence of ethical approval by an appropriate body? | Do the conclusions drawing in the research report flow from the analysis, or interpretation, of the data? |  |
| Austin et al., 2021 | [22] | YES | YES | YES | YES | YES | NO | YES | YES | NO | YES | 8/10 |
| Gribben et al., 2023 | [23] | NO | YES | YES | YES | YES | NO | YES | YES | YES | YES | 8/10 |
| Kong & Ganapathy, 2022 | [24] | YES | YES | YES | NO | YES | YES | NO | YES | NO | YES | 7/10 |
| Missouridou et al., 2021 | [10] | YES | YES | YES | YES | YES | YES | NO | YES | YES | YES | 9/10 |
| Moreno-Mulet et al., 2021 | [11] | YES | YES | YES | YES | YES | NO | NO | YES | YES | YES | 8/10 |

**REFERENCES (AMA 11^th^ ed.)**

1. Amir K, Okalo P. Frontline nurses’ compassion fatigue and associated predictive factors during the second wave of Covid-19

in Kampala, Uganda. *Nurs Open*. 2022;9(5):2390-2396. 10.1002/nop2.1253.

1. Carmassi C, Dell’Oste V, Bertelloni CA, Pedrinelli V, Barberi FM, Malacarne P, Dell’Osso L. Gender and occupational role

differences in work-related post-traumatic stress symptoms, burnout, and global functioning in emergency healthcare workers. *Intens Crit Care Nur*. 2022;69:103154. 10.1016/j.iccn.2021.103154.

1. Cheng J, Cui J, Yu W, Kang H, Tian Y, Jiang X. Factors influencing nurses’ behavioral intention toward caring for Covid-19

patients on mechanical ventilation: A cross-sectional study. *PloS One*. 2021;16(11):e0259658. 10.1371/journal.pone.0259658.

1. Cuartero-Castañer ML, Hidalgo-Andrade P, Cañas-Lerna AJ. Professional quality of life, engagement, and self-care in

healthcare professionals in Ecuador during the Covid-19 pandemic. *Healthcare (Basel)*. 2021;9(5):515. 10.3390/healthcare9050515.

1. Hochwarter W, Jordan S, Kiewitz C, Liborius P, Lampaki A, Franczak J, Deng Y, Babalola MT, Khan AK. Losing

compassion for patients? The implications of Covid-19 on compassion fatigue and event-related post-traumatic stress disorder in nurses. *J Manag Psychol*. 2022;37(3):206-223. 10.1108/JMP-01-2021-0037.

1. Kase SM, Gribben JL, Guttmann, KF, Waldman ED, Weintraub AS. Compassion fatigue, burnout, and compassion

satisfaction in pediatric subspecialists during the SARS-CoV-2 pandemic. *Pediatr Res*. 2022;91(1):143-148. 10.1038/s41390-021-01635-y.

1. Kaya SD, Mehmet N, Safak K. Professional commitment, satisfaction and quality of life of nurses during the Covid-19

pandemic in Konya, Turkey. *Ethiop J Health Sci*. 2022;32(2):393-404. 10.4314.ejhs.v32i2.20.

1. Kottoor AS, Chacko N. Role of entrapment in relation between fear of Covid-19 and compassion fatigue among nurses. *Int J*

*Behav Sci*. 2022;15(4):250-255. 10.30491/IJBS.2022.288846.1573.

1. Labrague LJ, de los Santos JAA. Resilience as a mediator between compassion fatigue, nurses’ work outcomes, and quality of

care during the Covid-19 pandemic. *Appl Nurs Res*. 2021;61:151476. 10.1016/j.apnr.2021.151476.

1. Missouridou E, Mangoulia P, Pavlou V, Kritsotakis E, Stefanou E, Bibou P, Kelesi M, Fradelos EC. Wounded healers during

the Covid-19 syndemic: Compassion fatigue and compassion satisfaction among nursing care providers in Greece. *Perspect Psychiatr Care*. 2021;58(4):1421-1432. 10.1111/ppc.12946.

1. Moreno-Mulet C, Sansó N, Carrero-Planells A, López-Deflory C, Galiana L, García-Pazo P, Borràs-Mateu MM, Miró-Bonet

M. The impact of the Covid-19 pandemic on ICU healthcare professionals: A mixed methods study. *IJERPH*. 2021;18(7):9243. 10.3390/ijerph18179243.

1. Perez-Chacon M, Chacon A, Borda-Mas M, Avargues-Navarro ML. Sensory processing sensitivity and compassion

satisfaction as risk/protective factors from burnout and compassion fatigue in healthcare and education professionals. *IJERPH*. 2021;18(2):611. 10.3390/ijerph18020611.

1. Ramaci T, Barattucci M, Ledda C, Rapisarda V. Social stigma during Covid-19 and its impact on HCWs outcomes.

*Sustainability (Basel)*. 2020;12(9):3834-3846. 10.3390/su12093834.

1. Ruiz-Fernàndez MD, Ramos-Pichardo JD, Ibànez-Masero O, Cabrera-Troya J, Carmona-Rega MI, Ortega-Galàn ÁM.

Compassion fatigue, burnout, compassion satisfaction and perceived stress in healthcare professionals during the Covid-19 health crisis in Spain. *J Clin Nurs*. 2020;29(21-22):4321-4330. 10.1111.jocn.15469.

1. Ruiz-Fernàndez MD, Ramos-Pichardo JD, Ibànez-Masero O, Carmona-Rega MI, Sànchez-Ruiz MJ, Ortega-Galàn ÁM.

Professional quality of life, self-compassion, resilience, and empathy in healthcare professionals during Covid-19 crisis in Spain. *Res Nurs Health*. 2021;44(4):620-632. 10.1002/nur.22158.

1. Spiridigliozzi SJ. Exploring the relationship between faith and the experience of burnout, compassion fatigue, and compassion

satisfaction for hospice workers during a global pandemic: A multidisciplinary study. *Doctoral Dissertation*. Lynchburg, VA: Liberty University; 2022.

1. Stevenson MC, Schaefer CT, Ravipati VM. Covid-19 patient care predicts nurses’ parental burnout and child abuse: Mediating

effects of compassion fatigue. *Child Abuse Negl*. 2021;30:105458. 10.1016/j.chiabu.2021.105458.

1. Su PA, Lo MC, Wang CL, Yang PC, Chang CI, Huang MC, Huan MK, Cheng KI. The correlation between professional

quality of life and mental health outcomes among hospital personnel during the Covid-19 pandemic in Taiwan. *J Multidiscip Healthc*. 2021;14:3485-3495. 10/2147/JMDH.S330533.

1. Yilmaz A, Bay F, Erdem O, Ozkalp B. The professional quality of life for healthcare workers during the Covid-19 pandemic in

Turkey and the influencing factors. *Bezmialem Sci*. 2022;10(3):361-369. 10.14235/bas.galenos.2021.5837.

1. Zakeri, MA, Rahiminezhad E, Salehi F, Ganieh H, Dehghan M. Compassion satisfaction, compassion fatigue and hardiness

among nurses: A comparison before and during the Covid-19 outbreak. *Front Psychol*. 2021;12:815180. 10.3389/fpsyg.2021.815180.

1. Nishihara T, Ohashi A, Nakashima Y, Yamashita T, Hiyama K, Kuroiwa M. Compassion fatigue in a health care worker

treating Covid-19 patients: A case report. *BioPsychoSoc Med*. 2022;16(1):10. 10.1186/s13030-022-00239-0.

1. Austin EJ, Blacker A, Kalia I. “Watching the tsunami come”: A case study of female healthcare provider experiences during

the Covid-19 pandemic. *Appl Psychol Health Well-Being*. 2021;13(4):781-797. 10.1111/aphw.12269.

1. Gribben JL, Kase SM, Guttmann KF, Waldman ED, Weintraub AS. Impact of the SARS-CoV2 pandemic on pediatric

subspecialists’ well-being and perception of workplace value. *Pediatr Res*. 2023:1-7. 10.1038.s41390-023-02474-9.

1. Kong KYC, Ganapathy S. Are we in control of our demons? Understanding compassion satisfaction, compassion fatigue and

burnout in an Asian pediatric emergency department in a pandemic. *Pediatr Emerg Care*. 2022;32(3):e1058-e1062. 10.1097/PEC.0000000000002656.
